# Network Pharmacology and Molecular Docking Study on the Potential Mechanism of Yi-Qi-Huo-Xue-Tong-Luo Formula in Treating Diabetic Peripheral Neuropathy

**DOI:** 10.1155/2021/9941791

**Published:** 2021-05-28

**Authors:** Yixuan Lin, Chuqiao Shen, Fanjing Wang, Zhaohui Fang, Guoming Shen

**Affiliations:** ^1^Graduate School of Anhui University of Chinese Medicine, Hefei, Anhui, China; ^2^Department of Pharmacy, The First Affiliated Hospital of Anhui University of Traditional Chinese Medicine, Hefei, Anhui, China; ^3^Department of Endocrinology, The First Affiliated Hospital of Anhui University of Traditional Chinese Medicine, Hefei, Anhui, China; ^4^Anhui Academic of Traditional Chinese Medicine Diabetes Research Institute, Hefei, Anhui, China

## Abstract

**Objective:**

To investigate the potential mechanism of action of Yi-Qi-Huo-Xue-Tong-Luo formula (YQHXTLF) in the treatment of diabetic peripheral neuropathy (DPN).

**Methods:**

Network pharmacology and molecular docking techniques were used in this study. Firstly, the active ingredients and the corresponding targets of YQHXTLF were retrieved using the Traditional Chinese Medicine Systems Pharmacology (TCMSP) platform; subsequently, the targets related to DPN were retrieved using GeneCards, Online Mendelian Inheritance in Man (OMIM), Pharmgkb, Therapeutic Target Database (TTD) and Drugbank databases; the common targets of YQHXTLF and DPN were obtained by Venn diagram; afterwards, the “YQHXTLF Pharmacodynamic Component-DPN Target” regulatory network was visualized using Cytoscape 3.6.1 software, and Gene Ontology (GO) enrichment analysis and Kyoto Encyclopedia of Genes and Genomes (KEGG) pathway analysis were performed on the potential targets using R 3.6.3 software. Finally, molecular docking of the main chemical components in the PPI network with the core targets was verified by Autodock Vina software.

**Results:**

A total of 86 active ingredients and 229 targets in YQHXTLF were screened, and 81 active ingredients and 110 targets were identified to be closely related to diabetic peripheral neuropathy disease. PPI network mapping identified TP53, MAPK1, JUN, and STAT3 as possible core targets. KEGG pathway analysis showed that these targets are mostly involved in AGE-RAGE signaling pathway in diabetic complications, TNF signaling pathway, and MAPK signaling pathway. The molecular docking results showed that the main chemical components of YQHXTLF have a stable binding activity to the core pivotal targets.

**Conclusion:**

YQHXTLF may act on TP53, MAPK1, JUN, and STAT3 to regulate inflammatory response, apoptosis, or proliferation as a molecular mechanism for the treatment of diabetic peripheral neuropathy, reflecting its multitarget and multipathway action, and providing new ideas to further uncover its pharmacological basis and mechanism of action.

## 1. Introduction

Diabetic peripheral neuropathy (DPN) is one of the most common and serious microvascular complications of diabetes which is characterized by pain, sensory abnormalities, and loss of sensation [1, 2]. It has been shown that DPN affects approximately 40% to 60% of people with diabetes [3], and if not well treated, it increases the risk of disability and mortality [4]. Currently, there is no specific treatment for DPN in modern medicine, which consists mainly of improving metabolic disorders and pain management [5, 6]. With the continuous improvement in medical care and the increasing demand for health, the main issue facing us today is how to prevent and control the progression of DPN and improve the quality of survival of patients. Traditional Chinese Medicine (TCM) has a long history of treating diabetes mellitus and its complications [7, 8]. In TCM, DPN is often classified as “paralysis,” “impotence,” and “blood paralysis” [9]. It is often caused by prolonged thirst, depletion of Qi and blood, deficiency of both yin and yang, and loss of nourishment for the tendons and veins, resulting in coldness, numbness, and muscle atrophy [10, 11]. Treatment is mostly based on benefiting Qi and nourishing Yin, invigorating blood circulation and relieving pain, emphasizing the treatment of both the symptoms and the root cause, and overall regulation [12]. A large number of studies have shown that Chinese medicine is effective in treating DPN, significantly improving the clinical symptoms of patients and delaying the development of the disease, with few toxic side effects [13]. Therefore, exploring the regulatory mechanisms of TCM can help develop new therapeutic approaches to improve the treatment of DPN.

Yi-Qi-Huo-Xue-Tong-Luo formula (YQHXTLF) is an in-hospital preparation for the prevention and control of diabetic peripheral neuropathy at Anhui Provincial Hospital of Traditional Chinese Medicine. It consists of 7 Chinese herbal medicines: Astragalus (Huangqi), Radix Angelicae Sinensis (Danggui), Radix et Rhizoma Dioscoreae (Dihuang), Radix et Rhizoma Yanhusuo (Yanhusuo), Radix et Rhizoma Puerariae (Gegen), Radix et Rhizoma Chrysanthemum (Jixueteng), and Radix et Rhizoma Weilingensis (Weilingxian). The combination of all the herbs in this formula can treat both the symptoms and the root cause of pain by treating Qi and blood together, which can benefit Qi and invigorate blood, as well as promote circulation and relieve pain. Preliminary clinical studies have shown that this formula can alleviate abnormal skin sensation, numbness, and tingling in the limbs and improve motor nerve conduction speed and sensory nerve conduction speed in DPN patients [14]. Basic research found that this formula can reduce islet cell damage in db/db mice, improve blood flow in the sciatic nerve area, and promote the repair and regeneration of damaged nerves, and the mechanism of action may be related to the improvement of diabetic inflammatory lesions and inhibition of excessive activation of the MAPK signaling pathway [15]. However, due to the multicomponent and multitarget nature of the Chinese medicine formula, the exact mechanism of action of the formula is still unclear.

Network pharmacology is a new discipline involving the analysis of drug-target-disease network associations [16]. It provides a systematic approach to the analysis of complex drug mechanisms of action and potential disease interventions by identifying the core targets shared by drugs and diseases [17, 18]. Through the use of network pharmacology, we can not only explore the complex active molecular components and potential molecular targets in Chinese medicine formulations but also understand the molecular relationships between different components in a compound formula and between the components and complex diseases and extract possible pathways for drug interventions to target diseases [19]. With the rapid development of network pharmacology, the mechanisms of TCM in the treatment of many serious diseases have been successfully predicted, and the multitarget integrated prevention and treatment approach has been applied to cancer [20], arthritis [21], diabetes, and other diseases with certain results [22, 23].

**Figure 1:**
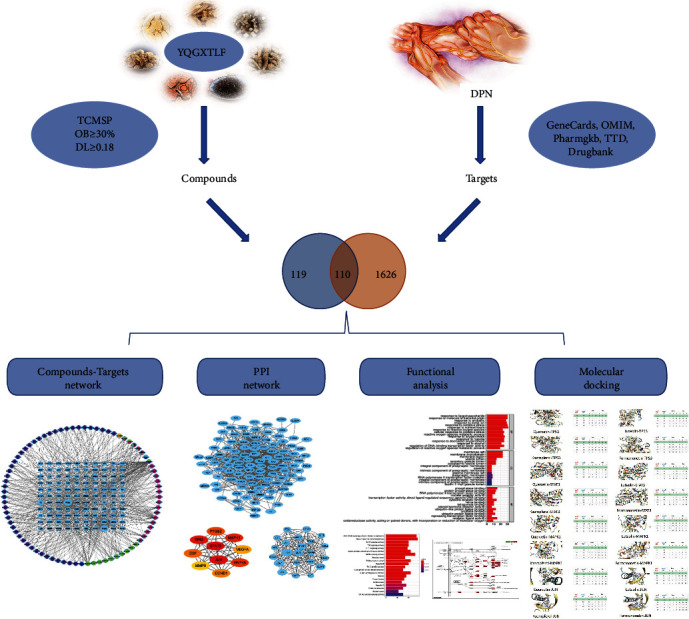
Workflow of the network pharmacology of YQHXTLF in the treatment of DPN. First, the effective active compounds of YQHXTLF were screened from the TCMSP. Relevant targets of DPN were summarized by searching databases. The intersection targets of compound targets and disease targets were obtained soon. Secondly, the interaction net between the compounds and the filtered targets was established. These key targets were analyzed by PPI analysis, functional analysis, and molecular docking verification. Finally, the key genes were used to find the biologic pathway and explain the therapeutic mechanism by network pharmacology analysis.

In this study, we use network pharmacology as a tool to further analyze the possible targets, molecular mechanisms, biological processes, and pathways of YQHXTLF for the treatment of DPN. We modeled the interrelationship between the targets of DPN and elucidated the synergistic mechanism between the active components of Chinese medicine, providing insights into the interrelationship and changes between Chinese medicine and diseases from the perspective of biological networks, which provided new possibilities and directions for the treatment of DPN. The flow chart of this study is shown in Figure 1.

## 2. Materials and Methods

### 2.1. Screening of Active Ingredients of YQHXTLF

The Traditional Chinese Medicine Systems Pharmacology (TCMSP, https://www.tcmspw.com) analysis platform [24] was used to screen the chemical components of YQHXTLF. TCMSP is a unique systemic pharmacology platform for Chinese herbal medicines that captures the relationship between drug, target, and disease. Oral bioavailability (OB) is one of the most important pharmacokinetic parameters in drug absorption, distribution, metabolism, and excretion (ADME) [25]. Drug-like properties (DL) refer to the similarity of a compound to a known drug. In drug development, drug-like studies are based on lead compounds, and drug-like molecules can be considered as high-quality lead compounds [26]. In this study, compounds with OB ≥ 30% and DL ≥ 0.18 were selected as potential active ingredients. The target information of these active ingredients was standardized by using the Uniprot database (https://www.uniprot.org/) with the species “homo sapiens.”

### 2.2. Exploring Potential DPN Targets

We searched several important databases with the keyword "diabetic peripheral neuropathy", including the GeneCards database (https://www.genecards.org/), the OMIM database (https://www.omim), the Pharmgkb database (https://www.pharmgkb.org/), the TTD database (http://bidd.nus.edu.sg/group/cjttd/), and the Drugbank data (https://www.drugbank.ca/). For the targets in GeneCards, only those with a score ≥ 10 were chosen. The targets obtained from the above four databases were then integrated to construct a DPN-related target set.

### 2.3. Constructing a Regulatory Network of Targets for the Treatment of DPN with YQHXTLF

The overlapping targets were considered to be the common targets of YQHXTLF and DPN. Cytoscape (version 3.6.1; https://www.cytoscape.org/) [27] was then used to visualize the complex relationships between the active chemical components and the potential target genes to construct a regulatory network of targets for the treatment of DPN with YQHXTLF. The layout tool was used to quantify the degree of each node; the larger the node in the network, the higher the degree value of the node.

### 2.4. Protein-Protein Interaction Analysis and Core Gene Screening

The intersecting gene targets of YQHXTLF and DPN were imported into the STRING protein interaction analysis platform, and the protein classification was set to “homo sapiens” with a maximum confidence level of ≥0.7, hiding the unlinked nodes in the network. Protein interaction network analysis was performed, and the TSV file was downloaded. Cytoscape 3.6.1 software was then imported to construct protein-protein interaction (PPI) network maps. The hub targets in the PPI network were screened using the cytoHubba plugin, with darker node colors representing higher scores [28]. The Molecular Complex Detection (MCODE) plugin was used to discover closely linked regions in the PPI network [29]. The score value of a module reflects how dense the module is to the surrounding nodes, with higher scores indicating that the nodes are becoming more important.

### 2.5. Gene Ontology and KEGG Pathway Enrichment Analysis

Gene Ontology (GO) functional analysis is mainly used to describe the function of gene targets, including biological process, cellular component, and molecular function. KEGG enrichment analysis can obtain the signal pathways enriched by the common targets of YQHXTLF and DPN. By using R 3.6.3 software-related R packages (Colorspace, Stringi, GGPLOt2, BiocManager, Dose, clusterProfiler, Enrichplot, Pathview), GO enrichment analysis and KEGG pathway enrichment analysis were conducted for the intersection target genes of YQHXTLF-DPN. According to Fisher's test, a *p* value < 0.05 and *q* value < 0.05 were considered statistically significant.

### 2.6. Molecular Docking Validation

Molecular docking validation is achieved through the CB-Dock server [30], a docking tool that predicts the binding site of a given protein and calculates the center and size using a novel curvature-based lumenal detection method. The 2D structure of the compound's small-molecule ligand was first obtained from the PubChem online database (https://pubchem.ncbi.nlm.nih.gov/) as the small-molecule ligand file for molecular docking. The core target protein receptor structure file was obtained from the PDB online database (https://www.rcsb.org). The CB-Dock software was used for molecular docking, and the score of the molecular docking complexes was calculated using the Vina program to evaluate their binding activity.

## 3. Results

### 3.1. Active Ingredients in YQHXTKF

The active ingredients were screened by searching the TCMSP database with a threshold value of OB ≥ 30% and DL ≥ 0 : 18. A total of 86 active ingredients derived from YQHXTLF were screened. Among them, 20 ingredients were from Astragalus, 2 from Radix Angelicae Sinensis, 4 from Radix Puerariae, 24 from Spatholobi Caulis, 2 from Rehmanniae Radix, 7 from Radix Clematidis, and 49 from Corydalis Rhizoma. The search for their corresponding targets showed that 86 compounds acted on 229 targets. The basic information of the 86 active compounds is shown in Table 1.

### 3.2. DPN-Related Gene Targets

Using the keyword “Diabetic peripheral neuropathy,” the GeneCards website screened 1521 genes related to diabetic peripheral neuropathy, the OMIM database screened 118, the Pharmgkb database retrieved 198, the TTD online website retrieved 32, and the Drugbank database retrieved 20. After removing duplicate targets and standardized gene names, a total of 1736 DPN potential action targets were screened.

### 3.3. Intersection of TQHXTLF and DPN Target

**Figure 2:**
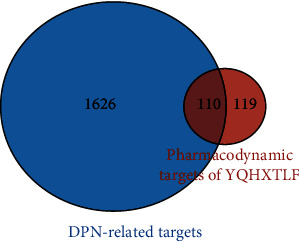
Venn diagram of targets of DPN treated by YQHXTLF.

The Hiplot research data visualization platform (https://hiplot.com.cn) was used to show the intersection of TQHXTLF and DPN targets, and Venn diagrams were drawn to obtain a total of 110 candidate targets for TQHXTLF and DPN, as shown in Figure 2.

### 3.4. Regulatory Network Analysis of YQHXTLF and DPN Targets

**Figure 3:**
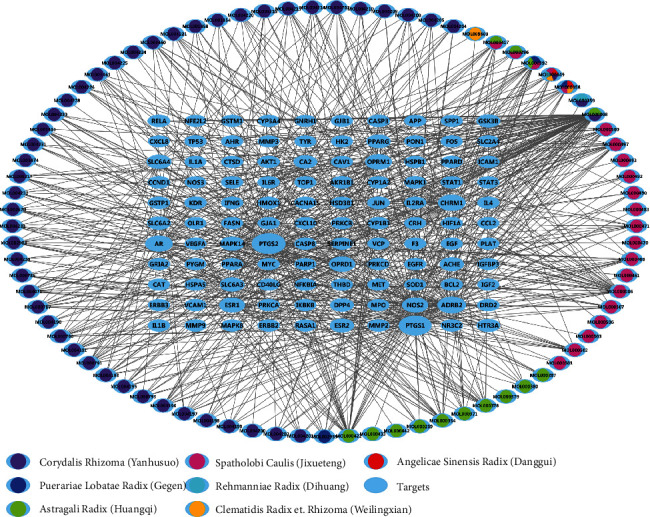
Construction of a target modulation network for DPN treatment with YQHXTLF. The blue ellipse represents the target site, the other coloured nodes represent the different herbal compounds, and the connecting lines represent the interaction between the compound and the target site.

The “YQHXTLF Pharmacodynamic Component-DPN Target” regulatory network was mapped by using Cytoscape 3.6.1 software to match the YQHXTLF pharmacodynamic components with the 110 action targets obtained from the screening. As shown in Figure 3, the network contained 191 nodes and 696 relationships. From the overall characteristics of the network, it can be found that among the 81 compounds in YQHXTLF, there is one compound corresponding to multiple targets and one target corresponding to multiple compounds; the larger the node, the larger the network value of the target. From the regulatory network, it can be seen that the chemical with the highest degree of connectivity is quercetin, which interacts with 75 targets, followed by luteolin, which interacts with 31 targets; kaempferol, which interacts with 28 targets; and formononetin, which interacts with 18 targets.

### 3.5. PPI Network Construction and Core Module Analysis

**Figure 4:**
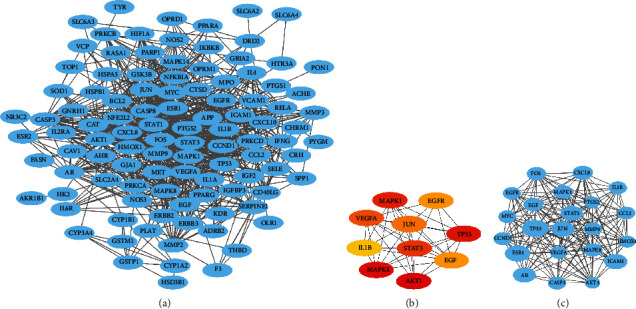
PPI network construction and core module: (a) YQHXTLF pharmacodynamic composition-DPN target PPI network; (b) top 10 hub target genes ranked by node degree; (c) the most significant modules analyzed by the MCODE plugin.

We imported common targets into the STRING 11.0 platform and visualized the PPI network using Cytoscape 3.6.1 software, as shown in Figure 4(a). 105 nodes and 797 edges were included in the network. Five targets did not interact with any other targets and were therefore not included in the PPI network. The cytoHubba plugin was applied to calculate the degree of degree connectivity for each target (Figure 4(b)). The top 10 hub target genes ranked by node degree were AKT1, MAPK8, TP53, MAPK1, STAT3, VEGFA, JUN, EGFR, and EGF. These target genes may play a key role in the network. The MCODE plugin was analyzed to filter out the most significant modules, with an MCODE score of 17.238, containing 22 nodes and 181 edges. The top ten targets in the MOCDE score were TP53, ESR1, ICAM1, JUN, AR, STAT3, CXCL8, MAPK1, and FOS (Figure 4(c)). The results showed that TP53, MAPK1, JUN, and STAT3 were the key targets under different algorithms.

### 3.6. Gene Ontology and KEGG Pathway Enrichment Analysis

**Figure 5:**
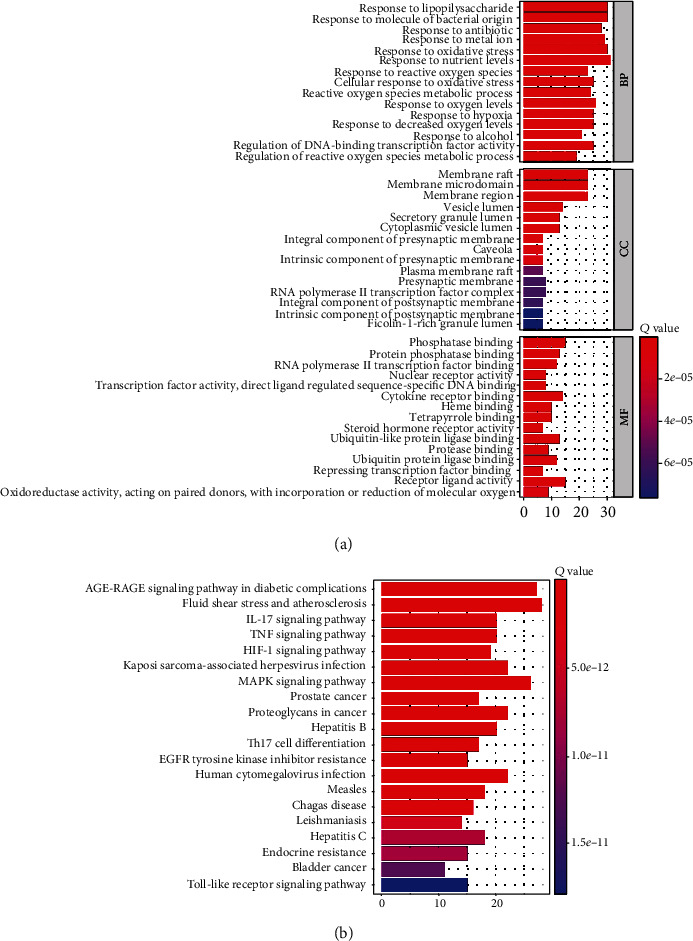
GO and KEGG pathway enrichment analysis: (a) the top 15 significantly enriched GO terms of BP, CC, and MF; (b) the top 20 significantly enriched KEGG pathways.

We intercepted the first 15 terms from the smallest to the largest according to the adj. *p* value. The results of the biological process analysis showed that the intersection targets were mostly enriched in response to lipopolysaccharide, response to molecule of bacterial origin, response to antibiotic, response to metal ion response to oxidative stress, and response to nutrient levels. The results of cell composition showed that the intersection targets were mostly enriched in membrane raft, membrane microdomain, membrane region, vesicle lumen, secretory granule lumen, cytoplasmic vesicle lumen, etc. Molecular functions mainly include phosphatase binding, protein phosphatase binding, RNA polymerase II transcription factor binding, nuclear receptor activity, transcription factor activity, direct ligand regulated sequence-specific DNA binding, and cytokine receptor binding, as shown in Figure 5(a).

We intercepted the top 20 KEGG pathways from the smallest to the largest based on the *p* value. The analysis showed that these targets were mostly enriched in the AGE-RAGE signaling pathway in diabetic complications, fluid shear stress and atherosclerosis, TNF signaling pathway, MAPK signaling pathway, etc., as shown in Figure 5(b).

### 3.7. Molecular Docking Validation

**Figure 6:**
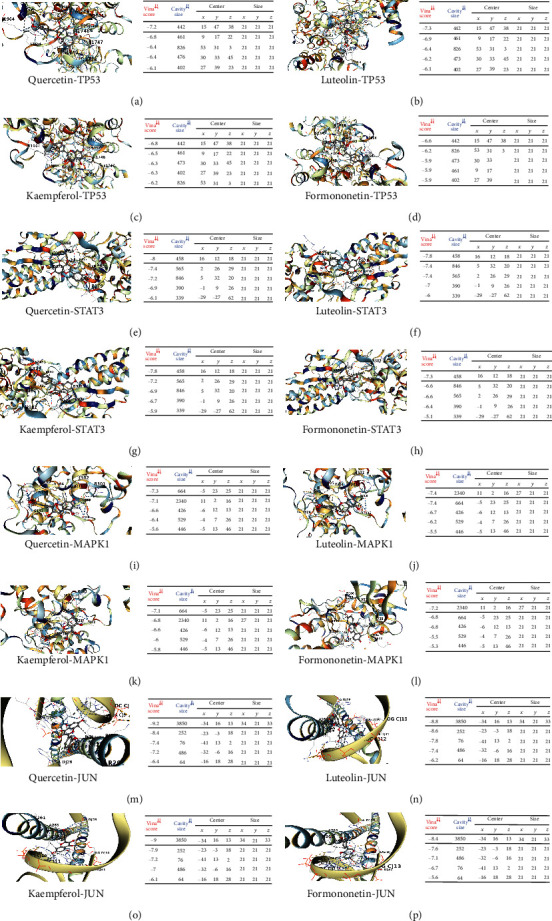
The docking model diagram of the active ingredient of the drug and the core target molecule: (a–d) the action mode of TP53 and quercetin, luteolin, kaempferol, and formononetin; (e–h) the action mode of STAT3 and quercetin, luteolin, kaempferol, and formononetin; (i–l) the action mode of MAPK1 and quercetin, luteolin, kaempferol, and formononetin; (m–p) the action mode of JUN and quercetin, luteolin, kaempferol, and formononetin.

The four active ingredients quercetin, luteolin, kaempferol, and formononetin, which have a high number of targets in YQHXTKF, were selected for molecular docking validation with the core targets TP53, MAPK1, JUN, and STAT3 in the PPI network map. The binding energy (Vina score) was used to evaluate the bonding strength between the docked molecules, and the value of the Vina score indicated some binding activity between the proteins and the compounds. The smaller the binding energy (Vina score), the higher the affinity of the receptor and ligand and the more stable the binding of the compound to the target site. The binding energies of the four active ingredients and the corresponding target proteins were all less than -5.0, indicating good docking and high binding activity [31]. Molecular docking simulations showed a stable point docking structure for the binding of small-molecule ligands and protein receptors (Figure 6).

## 4. Discussion

In this study, we used network pharmacology and molecular docking techniques to analyze the potential molecular biological mechanisms of YQHXTLF for the treatment of DPN. We firstly screened the active ingredients of YQHXTLF using the criteria of OB ≥ 30% and DL ≥ 0.18 and obtained 94 compounds, 229 potential targets, and 110 targets overlapping with DPN to construct a compound-disease target regulatory network. Through the network analysis, 81 components in this prescription may act on 110 targets to exert the therapeutic effect. These identified compounds, particularly the four compounds including quercetin, luteolin, kaempferol, and formononetin, were linked to more than 10 targets, indicating that these compounds might play a vital role in the process of DPN treatment. Quercetin is a flavonoid which is widely found in nature and has been proved to have a great effect on antibacterial, anti-inflammatory, and antiallergic therapy [32]. Quercetin reduces neuronal loss and inhibits neuronal apoptosis by improving neurotrophic factor levels [33, 34]. It has been reported that quercetin exerts neuroprotective effects on DRG neuronal cells in a high-glucose environment, possibly by activating Nrf-2/HO-1 and inhibiting NF-*κ*B to reduce apoptosis [35]. Quercetin also has neuroprotective effects in diabetic peripheral neuropathy by inducing autophagy to reduce the damage to neuronal cells from high glucose and improving the antioxidant status [36, 37]. Luteolin significantly upregulates the protein levels of NRF2 and HO-1 in diabetic nerves and improved nerve conduction velocity and nerve blood flow [38]. Luteolin has been shown to improve blood glucose, glycosylation, insulin, and HOMR-IR levels in diabetic model mice, with positive effects against diabetes and its complications [39]. Kaempferol has excellent antioxidant properties and can correct hyperglycemia in DM rats by regulating oxidative stress and reducing AGE accumulation, thus preventing the risk of complications [40, 41]. It reduces the expression of IL-1*β* and TNF-*α*, thereby inhibiting the neuroimmune activation of microglia and alleviating the progression of diabetic neuropathy [42]. Formononetin is an isoflavone that is known to prevent and slow the progression of long-term diabetic complications by reducing hyperglycemia and highlighting neuroprotective effects [43]. Formononetin protects diabetic animals from hyperglycemia-induced neuronal damage by increasing the expression of SIRT1 and NGF in neural tissue [44, 45]. Therefore, quercetin, luteolin, kaempferol, and formononetin may be the most important components of YQHXTLF for the treatment of DPN, as shown in Table 2.

**Figure 7:**
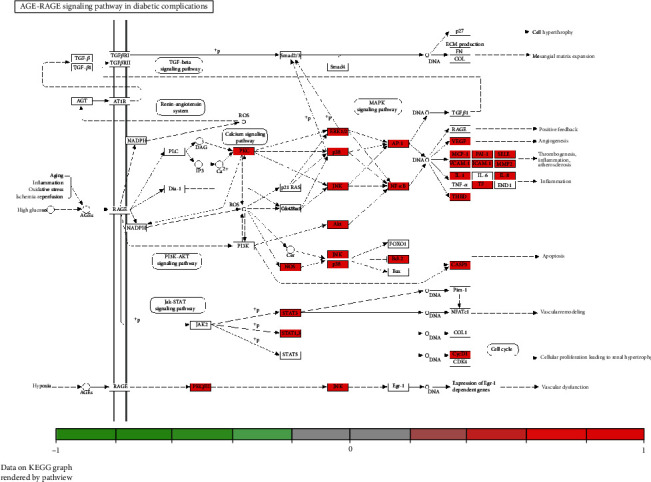
Pathway map of YQHXTLF in the treatment of DPN. AGE/RAGE signaling causes activation of multiple intracellular signaling pathways involving NADPH oxidase, protein kinase C (PKC), and MAPKs and promotes the expression of multiple proinflammatory cytokines, leading to segmental demyelination of peripheral neurons.

The exact etiology of DPN is still unclear, and its pathogenesis may be related to mitochondrial dysfunction and oxidative stress, polyol pathway activation, advanced glycosylation end products, and endoplasmic reticulum stress due to prolonged and severe hyperglycaemia [46, 47]. In addition, metabolic inflammation, neurotrophic vasculopathy, insulin resistance, and neurotrophic factors are all involved, creating a complex and interrelated pathogenesis [48]. Studies have shown that elevated levels of RAGE expression have been found in skin biopsy specimens from DPN patients [49]. Advanced glycosylation end products (AGEs) are a complex group of compounds. The primary receptor for AGEs (RAGE or AGER), which belongs to the immunoglobulin superfamily, has been described as a pattern recognition receptor [50]. AGE/RAGE signaling causes activation of multiple intracellular signaling pathways involving NADPH oxidase, protein kinase C (PKC), and MAPKs [51] and promotes the expression of multiple proinflammatory cytokines, leading to segmental demyelination of peripheral neurons (Figure 7). The interaction between AGE and RAGE leads to increased diacylglycerol (DAG) synthesis and excessive activation of PKC, which increases NF-*κ*B and tissue-type fibrinogen activator inhibitor-1 (PAI-1) expression, further activating inflammatory factors, transforming growth factor-*β* (TGF-*β*), and vascular endothelial growth factor (VEGF), leading to altered vascular function and peripheral neurological microangiopathy [52, 53]. Numerous studies have shown that AGEs bound to their receptors rapidly activate NADPH oxidase, increasing the level of mitochondrial oxidative stress, generating large amounts of ROS, and promoting a signal transduction cascade to induce apoptosis [54, 55]. STAT3 and MAPK pathways are important signaling pathways involved in inflammatory responses, cell proliferation, and apoptosis. STAT3, signal transducer and activator of transcription 3, a member of the signal transducer and activator of transcription family, is closely associated with central cell growth, proliferation and survival, and immune response [56]. Activated STAT3 can be implicated in the activity of downstream mediators such as p27kip1, P16INK4A, and p21kip1 proteins, which regulate cell growth, differentiation, and angiogenesis and are involved in the pathogenesis of diabetes [57]. MAPK is a mitogen-activated protein kinase involved in a variety of cellular functions, including cell proliferation, differentiation, and migration [58]. MAPKs cause neuronal apoptosis, impaired neuronal regeneration, and neuropathy through the direct action of glucose and glucose-induced oxidative stress [59]. Tumor necrosis factor-*α* (TNF-*α*) is a proinflammatory factor involved in peripheral nerve injury. It stimulates monocytes and endothelial cells to secrete IL-1*β* and IL-6 and other inflammatory factors, which have toxic effects on neurons and glial cells and lead to demyelination [60]. TNF-*α* inhibits nitric oxide synthase (NOS) activity in vascular endothelial cells, resulting in reduced NO-induced vasodilation, which leads to endothelial dysfunction and neurotrophic vascular damage and induces neuropathy [61]. In addition, TNF-*α* can activate the c-jun amino-terminal kinase JNK signaling pathway, leading to apoptosis [62]. TP53, cellular tumor antigen p53, is a stress-sensitive transcription factor responsible for controlling cell survival and death to prevent tumor formation [63]. TP53 has been found to inhibit glycolysis and to be involved in oxidative stress, the TP53 gene polymorphism marker Pro72Arg has been associated with DPN pathogenesis [64], and TP53 serum levels are significantly increased in patients with T2DM [65], so it is hypothesized that TP53 may play an important role in metabolic diseases such as diabetes. The molecular docking results showed that the active ingredients of the key compounds in YQHXTLF were able to bind stably to TP53, MAPK1, JUN, and STAT3. Thus, these results also confirm that our screened targets are consistent with literature reports, suggesting that YQHXTLF can play a therapeutic role in DPN by regulating apoptosis or proliferation and mediating inflammatory responses or oxidative stress.

## 5. Conclusion

In summary, this study analyzed the potential molecular biological mechanisms of YQHXTLF in the treatment of DPN throughnetwork pharmacology and molecular docking approach. The results showed that the active components of YQHXTLF in DPN treatment were composed of 81 compounds, among which quercetin, luteolin, kaempferol, and formononetin were the important active components. Moreover, a total of 110 target genes were screened, among which TP53, MAPK1, JUN, and STAT3 are possible core targets. The pathways involved in the treatment of DPN by YQHXTLF may be related to the AGE-RAGE signaling pathway, TNF signaling pathway, and MAPK signaling pathway, reflecting the multicomponent, multitarget, and multipathway biological properties of YQHXTLF. However, the network pharmacology is only a reasonable prediction of the mechanism of action of the herbal compound for the treatment of DPN based on the data mining perspective, which can provide a reference for the study of its therapeutic mechanism. We will be followed by animal and clinical experiments to validate the screened targets based on this prediction analysis, to provide more scientific evidence for its clinical application.

## Figures and Tables

**Table 1:** Detailed information of the 86 active compounds from YQHXTLF.

Mol Id	Mol name	Structure	OB (%)	DL
MOL000358	Beta-sitosterol	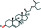	36.91	0.75
MOL000449	Stigmasterol	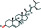	43.83	0.76
MOL000392	Formononetin	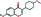	69.67	0.21
MOL002959	3′-Methoxydaidzein	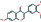	48.57	0.24
MOL003629	Daidzein-4,7-diglucoside	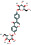	47.27	0.67
MOL000211	Mairin	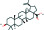	55.38	0.78
MOL000239	Jaranol	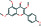	50.83	0.29
MOL000296	Hederagenin	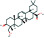	36.91	0.75
MOL000033	(3S,8S,9S,10R,13R,14S,17R)-10,13-Dimethyl-17-[(2R,5S)-5-propan-2-yloctan-2-yl]-2,3,4,7,8,9,11,12,14,15,16,17-dodecahydro-1H-cyclopenta[a]phenanthren-3-ol	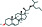	36.23	0.78
MOL000354	Isorhamnetin	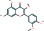	49.6	0.31
MOL000371	3,9-Di-O-methylnissolin	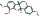	53.74	0.48
MOL000378	7-O-Methylisomucronulatol	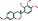	74.69	0.3
MOL000379	9,10-Dimethoxypterocarpane -3-O-*β*-D-glucoside	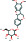	36.74	0.92
MOL000380	(6aR,11aR)-9,10-Dimethoxy-6a,11a-dihydro-6H-benzofurano[3,2-c]chromen-3-ol	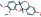	64.26	0.42
MOL000387	Bifendate	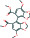	31.1	0.67
MOL000417	Calycosin	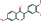	47.75	0.24
MOL000422	Kaempferol	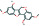	41.88	0.24
MOL000433	FA	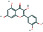	68.96	0.71
MOL000439	Isomucronulatol-7,2′-di-O-glucosiole	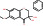	49.28	0.62
MOL000442	1,7-Dihydroxy-3,9-dimethoxy pterocarpene	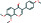	39.05	0.48
MOL000098	Quercetin	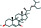	46.43	0.28
MOL000461	3,7-Dihydroxy-6-methoxy-dihydroflavonol	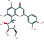	43.8	0.26
MOL000468	8-O-Methylreyusin	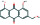	70.32	0.27
MOL000469	3-Hydroxystigmast-5-en-7-one	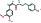	40.93	0.78
MOL000470	8-C-*α*-L-Arabinosylluteolin	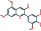	35.54	0.66
MOL000471	Aloe-emodin	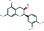	83.38	0.24
MOL000483	(Z)-3-(4-Hydroxy-3-methoxy-phenyl)-N-[2-(4-hydroxyphenyl)ethyl]acrylamide	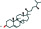	118.35	0.26
MOL000490	Petunidin	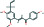	30.05	0.31
MOL000492	(+)-Catechin	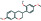	54.83	0.24
MOL000493	Campesterol	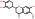	37.58	0.71
MOL000497	Licochalcone a	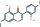	40.79	0.29
MOL000500	Vestitol	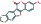	74.66	0.21
MOL000501	Consume close grain	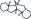	68.12	0.27
MOL000502	Cajinin	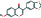	68.8	0.27
MOL000503	Medicagol	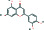	57.49	0.6
MOL000506	Lupinidine	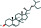	61.89	0.21
MOL000507	Psi-baptigenin	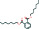	70.12	0.31
MOL000006	Luteolin	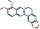	36.16	0.25
MOL000359	Sitosterol	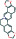	36.91	0.75
MOL005603	Diheptyl phthalate	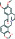	42.26	0.31
MOL001454	Berberine	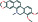	36.86	0.78
MOL001458	Coptisine	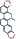	30.67	0.86
MOL001460	Cryptopine	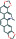	78.74	0.72
MOL001461	Dihydrochelerythrine	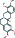	32.73	0.81
MOL001463	Dihydrosanguinarine	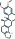	59.31	0.86
MOL001474	Sanguinarine	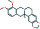	37.81	0.86
MOL000217	(S)-Scoulerine	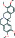	32.28	0.54
MOL002670	Cavidine	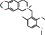	35.64	0.81
MOL002903	(R)-Canadine	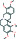	55.37	0.77
MOL004071	Hyndarine	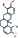	36.91	0.75
MOL004190	(-)-Alpha-N-methylcanadine	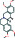	73.94	0.64
MOL004191	Capaurine	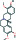	45.06	0.8
MOL004193	Clarkeanidine	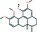	62.91	0.69
MOL004195	CORYDALINE	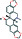	86.65	0.54
MOL004196	Corydalmine	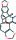	65.84	0.68
MOL004197	Corydine	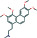	52.5	0.59
MOL004198	18797-79-0	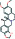	37.16	0.55
MOL004199	Corynoloxine	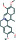	46.06	0.85
MOL004200	Methyl-[2-(3,4,6,7-tetramethoxy-1-phenanthryl)ethyl]amine	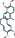	38.12	0.6
MOL004202	Dehydrocavidine	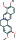	61.15	0.44
MOL004203	Dehydrocorybulbine	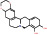	38.99	0.81
MOL004204	Dehydrocorydaline	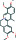	46.97	0.63
MOL004205	Dehydrocorydalmine	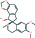	41.98	0.68
MOL004208	Demethylcorydalmatine	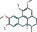	43.9	0.59
MOL004209	13-Methyldehydrocorydalmine	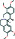	38.99	0.54
MOL004210	(1S,8′R)-6,7-Dimethoxy-2-methylspiro[3,4-dihydroisoquinoline-1,7′-6,8-dihydrocyclopenta[g][1,3]benzodioxole]-8′-ol	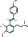	35.94	0.63
MOL004763	Izoteolin	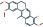	43.95	0.72
MOL004214	Isocorybulbine	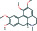	39.53	0.51
MOL004215	Leonticine	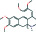	40.18	0.66
MOL004216	13-Methylpalmatrubine	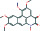	45.79	0.26
MOL004220	N-Methyllaurotetanine	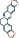	40.97	0.63
MOL004221	Norglaucing	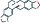	41.62	0.56
MOL004224	Pontevedrine	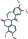	30.35	0.56
MOL004225	Pseudocoptisine	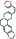	30.28	0.71
MOL004226	24240-05-9	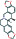	38.97	0.86
MOL004228	Saulatine	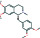	53.75	0.83
MOL004230	Stylopine	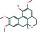	42.74	0.79
MOL004231	Tetrahydrocorysamine	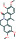	48.25	0.85
MOL004232	Tetrahydroprotopapaverine	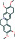	34.17	0.86
MOL004233	ST057701	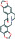	36.86	0.78
MOL004234	2,3,9,10-Tetramethoxy-13-methyl-5,6-dihydroisoquinolino[2,1-b]isoquinolin-8-one	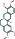	30.67	0.86
MOL000785	Palmatine	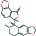	78.74	0.72
MOL000787	Fumarine	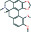	32.73	0.81
MOL000790	Isocorypalmine	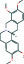	59.31	0.86
MOL000791	Bicuculline	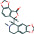	37.81	0.86
MOL000793	C09367	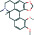	32.28	0.54

Mol Id: molecule Id; Mol name: molecule name; OB: oral bioavailability; DL: drug-like.

**Table 2:** The potential mechanisms of the four main compounds in the treatment of DPN.

Compound	Mechanism	Model	Reference
Quercetin	Increased BDNF, NGF, and Bcl-2, inhibited caspase-3	Diabetic rats	[33]
	Maintained the density of the general neuronal population, reduced the loss of interosseous neurons, antioxidant	Diabetic rats	[34]
	Activated the Nrf-2/HO-1 pathway, inhibited the NF-*κ*B pathway, and inhibited iNOS, COX-2, IL-6, and TNF-*α*	DRG cells	[35]
	Upregulated Beclin-1 and LC3 protein expression levels, increased cell proliferation, and upregulated autophagy	Schwann cells	[36]
	Reduced total cholesterol and TBARS levels, increased HDL-cholesterol, SOD, CAT, and GSH-Px activity	Db/db mice	[37]
Luteolin	Upregulated protein levels of Nrf2 and HO-1, improved nerve conduction velocity and nerve blood flow	Diabetic rats	[38]
	Improved the levels of blood glucose, HbA 1c, insulin, and HOMR-IR	KK-A^y^ mice	[39]
	Reduced mRNA expression of SREBP-1c, TNF-*α*		
Kaempferol	Regulated oxidative and nitrosative stress and reduced the formation of AGEs	Diabetic rats	[40]
	Reduced ROS production and inhibited caspase-3 activation	PC12 cells	[41]
	Reduction IL-1*β*, TNF-*α*, IC, and ROS and inhibited neuroimmune activation of microglia	Diabetic mice	[42]
Formononetin	Inhibited islet B cell apoptosis and promoted islet B cell regeneration, insulin secretion, hepatic glycogen synthesis, and hepatic glycolysis	Diabetic mice	[43]
	Controlled hyperglycemia and increased expression of SIRT1 and NGF	Diabetic rats	[44]
	Increased SIRT1 expression and reduced blood glucose	Diabetic rats	[45]

## Data Availability

We have presented all our main data in the form of figures and additional files. The data used to support the conclusions of this study are available from the authors.

## Supplementary Materials

Supplementary 1 Ingredients: potential active ingredients and action targets of the herbal compound were obtained through screening.

Supplementary 2 Diseases: DPN-related targets were obtained.
